# Effects of blending ratio variation on macronutrient compositions and sensory acceptability of dabi teff‐field pea‐based novel composite complementary flours

**DOI:** 10.1002/fsn3.3925

**Published:** 2023-12-26

**Authors:** Diriba Chewaka Tura, Tefera Belachew, Dessalegn Tamiru, Kalkidan Hassen Abate

**Affiliations:** ^1^ Department of Nutrition and Dietetics Institute of Health, Jimma University Jimma Ethiopia; ^2^ Department of Human Nutrition Wollega University Nekemte Ethiopia

**Keywords:** blending ratio variation, Dabi teff, macronutrients, novel complementary flour, sensory acceptability

## Abstract

The response of a mixed system is determined by the proportions of the various ingredients that add up to one, not by the combination's total amount. This research aimed at examining the effects of blending ratio variation on the macro‐composition and sensory acceptability of *dabi* teff‐field pea‐based novel composite complementary flours. Standard methods (AOAC, 2016, *Official Methods of Analysis of the Association of Official Analytical Chemists*) were used to determine macronutrients. The sensory attributes were evaluated using a 5‐point hedonic scale. The ingredients were constrained at 0%–30% for field pea, 20%–35% for *dabi* teff, and 5%–20% for maize, while the remaining were set constant at 5% linseed, 15% oats, and 25% barley. D‐optimal was used to examine the effects of blending ratio variation on the responses. All the responses were significantly different (*p* < .05) among the blends except for fat content, ranging from 14.58% to 17.21% for protein, 4.22% to 5.59% fat, 2.01% to 2.60% ash, 2.68% to 3.96% fiber, 68.08% to 70.76% utilizable carbohydrate, and 378.82 to 386.9 kcal/100 g gross energy. The sensory acceptability score ranged from 3.4 to 4.4. The linear model was significant (*p* < .05) and adequate to describe variation in moisture, protein, and ash contents. An increase in the ratio of field pea significantly increased (*p* < .05) protein, ash, fiber, and energy. The interactive effect between *dabi* teff and field pea significantly increased the sensory acceptability of the blends. These findings showed that varied proportions of the ingredients had a significant effect on the responses, and were used to develop a wholesome product to combat protein‐energy malnutrition among children.

## Graphical abstract

From this study, it can be summarized that the incorporation of field pea and dabi teff and their ratio variations in the complementary blends had demonstrated an increased energy and protein as well as sensory acceptability and could be suggested as sustainable food‐based strategy to combat the long term impact of protein‐energy malnutrition among children.

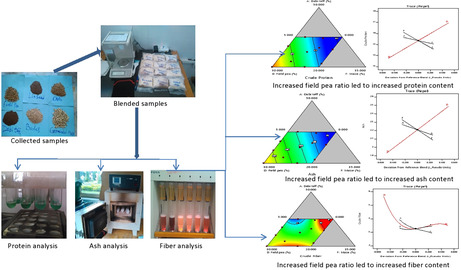

## 1 INTRODUCTION

Globally, there is an increasing tendency to shift from consuming animal‐source foods, especially highly processed meat products, to plant‐source foods because of the increased health risks associated with excess processed meat consumption (Mayer Labba et al., 2022). In the past, starting from the 1930s, there was a general belief that people needed to eat animal foods to obtain high‐quality protein, the ‘Great Protein Fiasco’, to prevent protein‐energy malnutrition in children, notably ‘Kwashiorkor in Africa’ (McLaren, 1974), but later, nutritionists recognized the relative importance of plant proteins and the need to formulate multi‐mixes of local food stuffs rather than commercial mixtures, where wheat/chickpea mixtures were successful by the WHO in 1966 (McLaren, 1974). In developing countries like Ethiopia, plant foods are the major staple diets and major food choices due to economic and religious reasons.

The nutritional and sensory qualities of plant‐based foods need to be improved, especially when to be used for child feeding. When the development of new food product targets infants and young children, special concern should be given to the sensory attributes of the product. This is because food acceptance by the target consumers is largely determined by sensory qualities rather than the nutritional value of the food (Gibson et al., 2006; Samuel et al., 2018). Foods derived from a variety of grains are more likely to contain several important nutrients and provide infants and young children an opportunity to experience a range of flavors, aromas, tastes, and textures, all of which are crucially important in building healthy eating habits in their later life (WHO, 2003).

Searching for different locally available, underutilized, affordable, and nutritious plant foods and blending them together (the food‐based approach) is the best option (Ruel & Levin, 2000; Usha et al., 2010) to get increased and multiple nutrients with enhanced sensory acceptability from their mixture and to provide a wholesome food product (containing all the essential amino acids and poly‐unsaturated fatty acids) as a main drive to prevent child protein‐energy malnutrition (FAO/WHO, 1991; McLaren, 1974).

The complementary feeding time (6–24 months of age) is one of the critical times in a child's life when the children transfer from breast milk to complementary feeding because the breast milk cannot satisfy the child's developing nutritional needs after 6 months (Rasane et al., 2015) and the infant's transfer from an ideal, nutritious, adequate, and uncontaminated breast milk to complementary foods (often regular family food) (Muhimbula & Issa‐Zacharia, 2010). FAO/WHO underscored that complementary foods should not replace (but complement) breastfeeding (Abebe et al., 2006; FAO/WHO, 1991).

Traditional complementary foods are characterized by low nutritional value (often monotonous) and are known to contain high bulk density, high viscosity, low protein, and low energy density. They are used in most developing countries, including Ethiopia. There are also suboptimal complementary feeding practices (Dewey & Adua Afarwuah, 2008; Gibson et al., 2000), which would result in elevated protein‐energy malnutrition in children. According to the Ethiopian Public Health Institute (EPHI) and the International Classification of Functioning, Disability and Health (ICF) survey, 37% of children under the age of 5 were stunted, 21% were underweight, 7% were wasted, and 2.9% were overweight (EPHI and ICF, 2019).

Recently, in Africa, cereal foods were complemented with locally available legumes such as soybeans, groundnuts, cowpeas, and pigeon peas, among others, as a protein source to improve the protein quality of the mix, and varied results were reported (Karakochuk et al., 2012; Muhimbula & Issa‐Zacharia, 2010).

Foods from multiple cereal‐legume blends are more likely to contain multiple or essential nutrients than foods from a single (monograin) which aroused the interest of nutritionists in examining the effects of mixing different grains at varied mixing proportions. Thus, it was hypothesized that blending different cereals and legumes, which are expected to be rich in nutrients at varied ratios, accompanied by proper processing techniques, would be promising to provide increased macronutrients with improved sensory acceptability.

*Dabi* teff is the ‘afaan oromoo’ language name for an early‐maturing variety of dark red teff. Due to its early maturing property, *dabi* teff is harvested twice within one rainy season (at early rainfall called “*daabi* gannoo” and late rainfall called “*daabi* birraa”) (Tura et al., 2023). The rural elderly people in particular and the consumers in general value dabi teff as a medicinal food, and there are many social beliefs regarding the nutritional and health benefits of this crop (Tura et al., 2023). Hence, in this study, the effects of blending ratio variation on macronutrient compositions and sensory acceptability of pre‐processed *dabi* teff‐field pea‐based novel composite complementary flours were examined to develop wholesome composite complementary flour to combat protein‐energy malnutrition among children.

## 2 METHODS AND MATERIALS

### 2.1 Sample collection

The food crops, viz., dabi teff *(Eragrostis teff (Zucc.) farmer variety*), maize (*Zea mays L*.), barley (*Hordeum vulgare*), white field pea (*Pisum sativum*), oats *(Avena sativa*), and linseed (*Linum usitatissimum*), were purchased from the open market of Nedjo town, Oromia, Ethiopia, which is located 575 km to the west from Addis Ababa, where Nedjo district is the potential *dabi* teff growing area. 3 kg of each apparently uninfected crop sample were purchased, and all were taken to the Ethiopian Institute of Agricultural Research, Food Science and Nutrition laboratory for further treatment after coding and packing them separately in polyethylene bags. All laboratory analysis of macronutrient contents and sensory evaluation was conducted in the stated laboratory, which was certified by the International Organization for Standardization (ISO‐17025:2017) by the International Laboratory Accreditation Cooperation (ILAC).

### 2.2 Sample processing

A number of controlled processing techniques were applied to the collected crop samples. In brief, to remove chaff, straw, dust, and other extraneous materials, the *dabi* teff sample was manually cleaned by winnowing, washed with tap water, and sundried. The other cereals (maize, barley, and oats) and the legumes (field pea and linseed) underwent individual processing by being sorted out from sands, sticks, stones, and defective seeds. Later, they were washed and sundried for 2 days at 27°C. *Dabi* teff was made into whole‐seed milled flour because of the small size of its seeds, which could be the reason for the higher nutrient contents of the crop. Barley and oats samples were soaked in clean tap water for 2 h, where the water used for soaking was drained off, and both crop samples were immediately decorticated (while the seeds were still wet) using a wooden decorticator. Their hulls were then removed by winnowing.

The germination of maize seeds was adapted from the method previously described by Rasane et al. (2015) with little modification. Briefly, in order to achieve hydration, the maize grains were soaked in water (1:3 w/v) for 3 h. The water used was drained off, and the seeds were spread on a clean jute sack placed on a wooden platform and covered with another jute sack for germination at room temperature (25°C ± 2°C) for 72 h, where they stayed for this 72 h because Rasane et al. (2015) reported that the maximum amylase activity was obtained at 72 h of germination time. Water was sprayed every 12 h to keep the maize seeds humid (60% relative humidity). For the purpose of terminating the germination kinetics, the germinated seeds were transferred to aluminum trays and dried in an air oven at 40°C for 5 h after rinsing and draining for 10 min. The dried germinated sample was further roasted at 120 ± 5°C for 10 min and allowed to cool.

The crop samples, viz., barley, oats, field pea, and germinated maize, were minimally roasted in an oven at 120°C for 20 min until light brown and then cooled to room temperature (25 ± 2°C) as described by Rasane et al. (2015). To prevent over‐roasting and avoid the formation of an undesirable Maillard reaction that may lead to protein quality damage, the roasting process was carefully controlled. To condition the release of oil from the oil cells, the linseed sample was minimally cooked for 5 min at 90°C (Castro‐Alba et al., 2019) with a small amount of water and later sundried without draining the water used for cooking.

### 2.3 Flour preparation and handling

To obtain a smooth and consistent particle size, all six processed samples were milled into flour using a standard miller (Cyclotec 1093 sample mill, Foss Analytical, Sweden) and sieved through a 0.5 mm mesh sieve size. The flours were then packed in airtight, high‐density polyethylene bags (AACC, 2000), separately coded, and stored safely at room temperature until formulation.

### 2.4 Experimental design

#### 2.4.1 Blending the flours

The blended design matrix (Table 1) was obtained by running the Stat‐Ease Design‐Expert® software version 11 (Randomized Mixture Design, D‐Optimal, Minneapolis, USA, 2018). Defining the range of each component in the blends was based on three considerations, including, first, targeting to attain the FAO/WHO (1991) macronutrients recommendation, second, targeting the Ethiopian complementary feeding guideline (Federal Democratic Republic of Ethiopia, 2012), and, third, targeting the macronutrient composition results of individual components (Table 2).

**TABLE 1:** D‐optimal mixture design matrix, blend code, mixture ratio, control flour, and constraints with their limits.

Std. order	Design ID	Run order	Mixture components ratio (%)						Limits of mixture components			
Standard	ID	Blends code	X1	X2	X3	X4	X5	X6	Total	Constraints	Lower	Upper
2	0	B1	27.5	25.0	15.0	15.0	5.0	12.5	All	X1	20	35
7	10	B2	26.123	25.0	15.0	23.877	5.0	5.0	100	X2	25	25
10	4	B3	30.457	25.0	15.0	19.543	5.0	5.0		X3	15	15
11	5	B4	20.000	25.0	15.0	29.988	5.0	5.012		X4	0	30
1	2	B5	28.793	25.0	15.0	6.207	5.0	20.0		X5	5	5
9	3	B6	35.000	25.0	15.0	11.884	5.0	8.116		X6	5	20
8	1	B7	21.705	25.0	15.0	13.295	5.0	20.0				
4	7	B8	34.385	25.0	15.0	6.476	5.0	14.139				
5	8	B9	20.000	25.0	15.0	19.54	5.0	15.46				
3	6	B10	22.735	25.0	15.0	22.515	5.0	9.75				
6	9	B11	29.604	25.0	15.0	10.076	5.0	15.32				
		Control	0	80	0	0	0	20				

*Note*: X1, Dabi Teff; X2, Barley; X3, Oats; X4, Field Pea; X5, Lin Seed; X6, Germinated Maize, B1–B11, blend s Code for each blend.

Abbreviations: ID, design identity; Std, Standard.

**TABLE 2:** Macronutrients and energy values of the processed individual flours.

Individual flours	Proximate composition (%)						Gross energy (kcal/100 g)	PER (g/100 kcal)	PEL
	Moisture	Cru. protein	Crude fat	Total ash	Crude fiber	Ut. carbohydrate			
Dabi teff	9.03 ± 0.10^a^	10.74 ± 0.04^a^	3.94 ± 0.58^a^	3.96 ± 0.03^a^	3.76 ± 0.16^a^	68.57 ± 0.51^a^	352.70^a^	3.045	12.180
Roasted Barley	4.32 ± 0.33^b^	11.62 ± 0.03^b^	2.58 ± 0.43^a^	1.73 ± 0.09^b^	2.12 ± 0.07^b^	77.63 ± 0.06^b^	380.22^b^	3.056	12.224
Dehulled Oats	7.16 ± 0.06^c^	12.45 ± 0.06^bc^	5.69 ± 0.03^a^	1.92 ± 0.01^bc^	2.78 ± 0.07^c^	70.00 ± 0.09^a^	381.01^bc^	3.268	13.071
Roasted Field pea	5.80 ± 0.01^bc^	20.95 ± 0.02^d^	2.74 ± 0.01^a^	2.60 ± 0.03^d^	7.46 ± 0.07^d^	60.45 ± 0.08^c^	350.26^a^	5.981	23.925
Cooked Linseed	6.32 ± 0.25^ce^	20.57 ± 0.30^de^	36.08 ± 1.45^b^	3.45 ± 0.01^e^	5.34 ± 0.11^e^	28.24 ± 1.61^d^	519.96^d^	3.956	15.824
Germinated Maize	7.68 ± 1.13^ac^	8.58 ± 0.13^f^	4.61 ± 0.95^a^	1.42 ± 0.01^bf^	2.47 ± 0.03^bcf^	75.24 ± 0.03^be^	376.77^be^	2.277	9.109

*Note*: Values are means ± standard deviation of the triplicate determinations. The values in the same column followed by different superscript letters are significantly different at *p* < .05.

Abbreviations: Cru, crude; PEL, protein energy level; PER, protein energy ratio; Ut, utilizable.

The macronutrient composition analysis results of individual flours (Table 2) were recorded (customized) into Nutriurvey software (Version 2007) to estimate and define ranges of major components. By estimating the amount of a meal to be consumed by 1–3‐year‐old children to be 75 g (solid portion) per meal and adjusting for the required number of meals per day, several trials (iterations) were made to define ranges of the major components (*dabi* teff, field pea, and germinated maize) by entering range‐related amounts into the software combined with the constant components. Finally, the generated output was examined, where it showed the percentage fulfillment by the meal of the mix as compared to the recommended dietary allowance (RDA) to be 84%–152%, 90%–188%, 107%–195%, 41%–61% for energy, protein, carbohydrate, and fat (Table 3), which corresponds to 20%–35% of *dabi* teff, 0%–30% of field pea, 5%–20% of germinated maize, 25% of barley, 15% of oats, and 5% of linseed meal mixture, respectively, which were used as constraints for generating the blending matrix.

**TABLE 3:** Nutrisurvey analysis of the food records (customized) to define ranges of each component in the blends.

		Calculated energy and nutrient values of the formulated meal by Nutrisurvey								
Components	Proportion (%) (lower–upper)	Amount eaten range/day (g)	Energy range (kcal)	CHO range (g)	Protein range (g)	Fat range (g)	Iron range (mg)	Calcium range (mg)	Zinc range (mg)	Magnesium range (mg)
Dabi teff	20–35	45–79	158.7–278.7	30.9–54.2						
Roasted Barley	25	56	212.9	43.5						
Dehulled Oats	15	34	129.5	23.8						
Roasted Field pea	0–30	0–68	0–238.3	0–41.1						
Cooked Linseed	5	12	62.4	3.4						
Germinated Maize	5–20	11–45	41.4–169.5	8.3–33.9						
Analyzed value (range) for the formulated meal using our components			604.9–1091.5^a^	109.9–199.9 (73%–74%)	19–39.8 (13%–15%)^a^	10–14.8 (15%–12%)	49–83.6^a^	87.8–176.1	4–6.7^a^	183–320.3
Recommended value for 1–3 years old child as generated by the ‘Nutrisurvey’ software			718.3	102.5 (>55%)	21.2 (12%)	24.4 (<30%)	8	600	3	80
Percentage fulfillment from the formulated meal (range) (lower–upper) %			84–152^a^	107–195^a^	90–188^a^	41–61	612–1045^a^	15–29	133–225^a^	229%–400%

^a^ Showing the blended complementary flours would be promising to attain the FAO/WHO recommendations.

The six components were constrained to provide 11 experimental runs. The ratios in grams of individual components generated in each blend (experimental run) were carefully weighed on a digital balance gravimetrically and blended together. The blended flours were thoroughly mixed using an electrical blender for 3 min at 200 rpm to homogenize the flours. They were then packed and sealed in high‐density polyethylene bags and stored in a refrigerator at 4°C till analysis.

#### 2.4.2 Porridge preparation

The method outlined by Onabanjo et al. (2008) was used to prepare thick and consistent porridge by mothers acquainted with good cooking skills from all the blends and the control with slight modifications. Briefly, 300 g of the composite flour was mixed with 500 mL of clean tap water in a saucepan to make a slurry and put aside. 800 mL of the water was boiled in a stainless steel pan, and once the water reached boiling point, the previously prepared slurry was added to the boiled water and allowed to cook for 10–15 min on an electric stove with continuous stirring, then taken from the stove and allowed to cool at the serving temperature of around 40°C. The usual (commonly consumed cereal‐based complementary food in the study area) was used as a control constructed from 80% barley flour and 20% ungerminated maize flour in consultation with caretakers/mothers. All the prepared porridges were subjected to sensory evaluation as fresh as possible by mother panelists.

#### 2.4.3 Macronutrient compositions analysis

To determine the macro‐compositions of the complementary blends and the individual flours, the Association of Official Analytical Chemists (AOAC) (2016) modified methods were used. The moisture content was determined by an air convection drying oven (Model No. DHG‐9121A, Sweden) using the method described by 925.10, AOAC (2016) for 1 h at 130 ± 3°C. Crude protein content was determined by Kjeldahl (Kjeltec 8400, Auto Sample Systems, Foss Analytical, Sweden) using a nitrogen conversion factor of 6.25 following the official method 954.10, AOAC (2016).

The Soxhlet method (Soxtec 8000, Tecator Line, Foss Analytical, Sweden) was used to determine crude fat content by N‐Hexane extract according to the method number 2003.06, AOAC (2016). Ash content was determined using the combustion method in a box‐type muffle furnace (Model No.SX2‐4‐10GJ, China) at 550°C for 4 h, following method 923.03, AOAC (2016). Crude fiber content was determined by Fibertec 8000, Foss Analytical, Sweden, following official method 978.10, AOAC (2016). Utilizable carbohydrate was estimated by the difference: 100 − (% Moisture +% Crude protein + % Crude fat + % Crude fiber + % Ash) (Agza et al., 2018; Ayele et al., 2021). The gross energy (kcal/100 g) of individuals and blended flours was computed based on FAO/WHO (1985) recently amended 2021 codex guidelines by multiplying the values obtained for energy‐yielding nutrients (crude protein, crude fat, and utilizable carbohydrate) with Atwater conversion factors, where *E* (kcal/100 g) = [(% crude fat × 9) + (% crude proteins × 4) + (% utilizable carbohydrates × 4)].

#### 2.4.4 Sensory evaluation of the porridges

Fourty‐eight untrained healthy panelists comprising mothers/caretakers having babies between 6 and 24 months have participated to evaluate the sensory attributes, including color, aroma, taste, mouthfeel, and overall sensory acceptability, of each porridge sample. The panelists were requested to evaluate each sample after they were briefed about scoring a sensory attribute using a 5‐point hedonic scale representing 5 – like very much, 4 – like moderately, 3 – neither like nor dislike, 2 – dislike moderately, and 1 – dislike very much. Finally, the mean sensory attribute score was created for each respondent to represent a particular trait.

### 2.5 Statistical analysis and model evaluation

All the laboratory analysis results of the 11 experimental runs (Table 1) and the sensory evaluation results were subjected to ‘Sheffe’ polynomial mixture regression analysis using the Stat‐Ease Design‐Expert® software version 11 (D‐optimal mixtures design). Linear, quadratic, cubic, and special cubic models and interactive effects of the independent variables were fitted for evaluation of the response variables, namely macronutrient compositions of the flours and sensory acceptability (Table 4) of the porridge prepared from the blends. An analysis of variance (ANOVA) of the Design‐Expert was performed to fit and develop regression models (mathematical algorisms) to show the relationship between Xs' (individual linear, quadratic, cubic, and interactive effects of the components) and Ys' (the response variables) and to determine the goodness of fit (significance) of the models developed. These effects (relationships) were further verified by running the ‘Model Graphs’ test through ‘2D contour plots’ and ‘trace plots’ graphs.

**TABLE 4:** Models fitted for macronutrients and statistical outputs showing model significance and adequacy.

Model's *p*‐value (model terms)	Macro‐compositions							SE
	MC	Protein	Fat	Ash	Fiber	CHO	Energy	OA
Linear (x_1_, x_4_, x_6_)	0.0049 #**	0.0005 #***	0.334	0.0211#*	0.3442	0.1790	0.3108#^	0.2196
Quadratic (x_1_^2^, x_4_^2^, x_6_^2^)	0.3313	0.4721	0.0030#**	0.7957	0.9237	0.0545	0.8144	0.0977
Special Cubic	0.5132	0.5436	0.9767	0.8995	0.2875#^	0.4498	0.3075	0.9245
Cubic	0.3819	0.4444	0.8637	0.9694	0.6022	0.0483#*	0.7794	0.0452
Sp. Quartic vs Quadratic	0.1780	0.4675	0.5789	0.8310	0.3177	0.3527	0.5419	0.0311#*
Interaction terms								
x_1_x_4_	–	–	0.0102	–	0.5564	0.0560	–	0.3471
x_1_x_6_	–	–	0.0064	–	0.2962	0.1521	–	0.0319
x_4_x_6_	–	–	0.0009	–	0.5077	0.0406	–	0.5901
x_1_x_4_x_6_	–	–	–	–	0.2875	0.9808	–	0.0308
*F*‐statistics (ANOVA)								
Model *F*‐value	11.15	22.23	16.61	6.49	0.64	257.55	1.36	31.58
Lack‐of‐Fit	PE0	PE0	PE0	PE0	PE0	PE0	PE0	PE0
*R* ^2^	0.75	0.8487	0.9432	0.6188	0.4899	0.9996	0.2534	0.9921
*R* ^2^%	75	84.87	94.32	61.88	48.99	99.96	25.34	99.21
ADP	8.53	12.961	12.72	6.979	2.856	55.029	2.76	19.95
Model	0.0049*	0.0005*	0.0039*	0.0211*	0.7025^	0.0483*	0.3108^	0.0311*

*Note*: #‐suggested, #*, #** and #***‐model is suggested and significant at *p* < .05, at *p* < .01 and at *p* < .001, respectively, #^ ‐suggested and not significant, * and ^ model is significant and not significant, respectively, x_1_, dabi teff; x_4_, field pea; x_6_,germinated maize.

Abbreviations: ADP, adequate precision; CHO, carbohydrate; MC, moisture content; OA, overall acceptability; PE0, pure error zero; *R* ^2^%, coefficient of determination; SE, sensory evaluation; Sp., special; vs, versus.

Linear and polynomial regression models were judged (verified) to be adequate and significant using the *F*‐statistic at a probability (*p*) of .05, .01, and .001 and the coefficient of determination *R* ^2^. The closer the *R* ^2^ value is to unity (1), the better the model fits the actual data, ensuring satisfactory fitted models that are adequate to specify the correct relationship between response (Ys') and independent variables (Xs') (Nahemiah et al., 2016). The normality and constant variance assumptions of the error terms were checked to determine whether a model meets the assumptions of the analysis. Additionally, one‐way ANOVA of SPSS (IBM version 24, Chicago, USA) was used to declare statistically significant differences between the blends and compare them to the control as well as the Cerifam® faffa flour (the popular commercial complementary flour in Ethiopia). All the data collected were in triplicate, except for the sensory evaluation. Levene's test was used to check the equal variance assumptions (*p* > .05 should be non‐significant). The Tukey honestly significant difference (HSD) post hoc test was used for the mean difference separation test, and the significant differences were declared at *p* < .05.

## 3 RESULTS

### 3.1 Model fitting and testing model adequacy

The fitted models were found to be adequate and significant for most response variables based on the *F*‐statistic (the ANOVA regression outputs), the *p*‐value, and the coefficient of determinations. Normality and constant variance assumptions were fulfilled.

Table 4 showed that the linear models were adequately fitted for moisture, protein, and ash contents and significant at *p*‐values of <.01, <.001, and <.05. This shows that x_1_, x_4_, and x_6_ (*dabi* teff, field pea, and germinated maize) were the significant model terms for these compositions. This means changes in moisture, protein, and ash can adequately be described by the linear models (adequate predictive power) as a function of the component ratio variations in the blends, where the regression equation is shown in Table 5. The quadratic model was adequately fitted for fat and significant at a *p*‐value of <.05. This shows that the linear model terms (x_1_, x_4_, and x_6_) and the interactive model terms (x_1_x_4,_ x_1_x_6_, and x_4_x_6_) were significant model terms for fat content. The linear model terms (*dabi* teff, field pea, and germinated maize) had shown a synergetic effect, whereas the interactive model terms (interactive effects) (*dabi* teff by field pea, *dabi* teff by germinated maize, and field pea by germinated maize) had shown a significant antagonistic effect on fat content in the blends at a *p*‐value <.05, which was also shown in Table 5 with the repression equation for fat.

**TABLE 5:** ANOVA regression models showing the effects of the independent variables (Xs') on dependent variables (Ys') in the blends.

Dependent variables (Ys')	Regression models with Xs' (actual equations)	Model
Moisture (Y_1_)	0.129969 x_1_ + 0.056327 x_4_ + 0.0406071 x_6_	Linear
Crude Protein (Y_2_)	0.267207 x_1_ + 0.350487 x_4_+ 0.219951 x_6_	Linear
Crude Fat (Y_3_)	0.195395 x_1_ + 0.228208 x_4_+ 0.508282 x_6_‐0.007827 x_1_x_4_–0.0146967 x_1_x_6_–0.013247 x_4_x_6_	Quadratic
Ash (Y_4_)	0.037258 x_1_+ 0.052819 x_4_+ 0.031447 x_6_	Linear
Crude Fiber (Y_5_)	−0.077218 x_1_–0.12638 x_4_–0.386782 x_6_ + 0.012573 x_1_x_4_ + 0.023008x_1_x_6_ + 0.036897 x_4_x_6_–0.001532 x_1_x_4_x_6_	Special Cubic
Utilizable Carbohydrate (Y_6_)	7.08547 x_1_–1.53572 x_4_–29.974 x_6_–0.0920234 x_1_x_4_ + 0.872668 x_1_x_6_ + 0.741828 x_4_x_6_ + 1.03834e‐05 x_1_x_4_x_6_–0.00550154 x_1_x_4_ (x_1_−x_4_) ‐ 0.0186895 x_1_x_6_ (x_1_−x_6_) − 0.00724686 x_4_x_6_ (x_4_−x_6_)	Cubic
Gross Energy (Y_7_)	6.88819 x_1_ + 6.91064 x_4_ + 7.13471 x_6_	Linear
Overall Acceptability (Y_8_)	2.90276 x_1_ + 4.00009 x_4_ + 4.02192 x_6_ + 2.62446 x_1_x_4_–14.8958 x_1_x_6_ + 1.18348 x_4_x_6_ + 140.849 x_1_^2^x_4_x_6_–51.8023 x_1_x_4_^2^x_6_ + 42.694 x_1_x_4_x_6_^2^	Special quartic

*Note*: x_1_, dabi teff, x_4_, field pea, x_6_, germinated maize.

On the other hand, a cubic model was significantly fitted for carbohydrates and significant at *p*‐values of <.05, where the linear model terms (x_1_, x_4_, x_6_) and the interactive model terms x_1_x_6_, x_4_x_6_ (x_1_−x_6_), and x_4_x_6_ (x_4_−x_6_) were the significant model terms for carbohydrate at P‐values less than 0.05. Meaningful changes in carbohydrates can be described by the linear, quadratic, and cubic model terms as a function of the component ratio variations in the blends. x_1_ (*dabi* teff), x_1_x_6_ (dabi teff by germinated maize), and x_4_x_6_ (field pea by germinated maize) had a synergetic effect on the carbohydrate, whereas x_4_, x_6_, and x_1_x_4_ had shown an antagonistic effect on carbohydrate content, as shown in Table 5 with the regression equation for carbohydrate. Likewise, the special quartic model was significantly fitted for overall sensory acceptability and was significant at a *p* value of .05.

Special cubic and linear models were suggested by the software for fiber and energy contents, respectively, but the models were non‐significant (*p*‐values >.05), with no predictive power in describing changes in fiber and energy content as a function of component ratio variations in the blends (regression equation is found in Table 5).

The predictive regression models developed for the relationship between the independent variables (Xs') and the dependent variables (Ys') in terms of macronutrient compositions and sensory acceptability of the blends were presented in Table 5 for moisture, crude protein, crude fat, ash, utilizable carbohydrate, gross energy, and overall sensory acceptability, respectively. The coefficients with a single factor (linear model terms) (X_1_, X_4_, and X_6_) represent the independent effect of a particular determinant variable, while the coefficients with two factors (X_1_X_4_, X_1_X_6_, and X_4_X_6_) represent the interaction between the factors (interactive model terms). A positive sign in front of the regression model terms (mathematical algorism) is an indication of a synergetic relationship, while a negative sign indicates an antagonistic relationship.

### 3.2 Effects of blending ratio variation on macronutrient compositions of the composite complementary flours

The response of a mixture system is determined by the ratio variation of the various ingredients that add up to one, not by the combination's total amount. In this study, the mean and standard deviation of energy and macronutrient composition of the blended complementary flours were summarized in Table 6. Mean difference separation results showed that all the macro‐compositions were significantly different (*p* < .05) among the blends (as affected by the component ratio variation) except for fat, where their content ranged from 4.41% to 5.74% for moisture, 14.58% to 17.21% protein, 4.22% to 5.59% fat, 2.01% to 2.60% ash, 2.68 to 3.96 fiber, 68.08% to 70.76% utilizable carbohydrate, and 378.82 to 386.9 kcal/100 g gross energy, respectively. These results were within the acceptable critical limits set by FAO/WHO (1991) guidelines, except for fat content at ≤5%, ≥15%, 10–25%, ≤3%, ≤5%, 64 ± 4% and 400 to 425 kcal/100 g for the respective composition parameters. The control flour contained 6.15%, 10.77%, 3.78%, 2.24%, 2.65%, and 74.41% and 374.74 kcal/100 g for the respective parameters mentioned (Table 6).

**TABLE 6:** Effects of blending ratios on energy and macro‐compositions of the blended fours and FAO/WHO (1991) recommendation.

Formulations	Proximate composition (%)						Gross energy (kcal/100 g)	Protein density (g/100 kcal)
	Moisture	Protein	Crude fat	Total ash	Crude fiber	Ut. Carbohydrate		
B1	4.58 ± 0.01^a^	15.08 ± 0.25^a^	4.22 ± 0.04^a^	2.31 ± 0.08^a^	3.68 ± 0.06^a^	70.13 ± 0.26^a^	378.82^a^	3.981
B2	4.61 ± 0.25^a^	16.51 ± 0.16^b^	4.75 ± 0.01^a^	2.45 ± 0.01^a^	3.56 ± 0.03^a^	68.12 ± 0.13^a^	381.27^a^	4.330
B3	5.74 ± 0.10^a^	15.93 ± 0.05^ab^	4.78 ± 0.06^a^	2.25 ± 0.06^a^	3.22 ± 0.01^b^	68.08 ± 0.04^a^	379.06^a^	4.203
B4	4.68 ± 0.54^a^	17.21 ± 0.17^bc^	5.14 ± 0.40^a^	2.60 ± 0.01^ab^	3.46 ± 0.04^a^	66.91 ± 0.29^b^	382.74^a^	4.496
B5	5.05 ± 0.16^a^	14.63 ± 0.02^a^	5.59 ± 0.57^a^	2.14 ± 0.01^ac^	3.82 ± 0.06^a^	68.77 ± 0.33^a^	383.91^b^	3.811
B6	5.52 ± 0.42^a^	15.27 ± 0.32^ab^	4.92 ± 0.69^a^	2.20 ± 0.11^a^	2.68 ± 0.01^c^	69.41 ± 048^a^	383^c^	3.987
B7	4.50 ± 0.01^ab^	15.17 ± 0.04^ab^	5.32 ± 0.06^a^	2.05 ± 0.02^ad^	3.74 ± 0.03^a^	69.22 ± 0.08^a^	385.44^d^	3.936
B8	5.45 ± 0.03^a^	14.65 ± 0.35^a^	5.30 ± 0.11^a^	2.09 ± 0.18^ae^	3.96 ± 0.06^ag^	68.55 ± 0.14^a^	380.5^a^	3.850
B9	4.41 ± 0.03^ab^	14.80 ± 0.09^a^	4.62 ± 0.33^a^	2.35 ± 0.02^a^	3.66 ± 0.06^a^	70.16 ± 0.19^a^	381.42^a^	3.880
B10	4.43 ± 0.04^ab^	16.53 ± 0.25^bd^	4.34 ± 0.12^a^	2.11 ± 0.03^af^	3.78 ± 0.06^a^	68.81 ± 0.08^a^	380.42^a^	4.345
B11	4.88 ± 0.04^a^	14.58 ± 0.04^a^	5.06 ± 0.19^a^	2.01 ± 0.19^ag^	2.71 ± 0.32^cd^	70.76 ± 0.03^a^	386.9^e^	3.768
Mean	4.8955	15.4873	4.913	2.233	3.479	68.993	382.135	4.053
Control	6.15 ± 0.82^c^	10.77 ± 0.52^e^	3.78 ± 0.79^a^	2.24 ± 0.01^a^	2.65 ± 0.03^ce^	74.41 ± 2.15c	374.74f	2.874
FAO/WHO	≤5	>15	10–25	≤3	≤5	64 ± 4	400–425	<5.5

*Note*: Values are means ± standard deviation of the triplicate determinations. The values in the same column followed by different superscript letters are significantly different at *p* < .05. The corresponding component ratio for each blend code (B1–B11) is well described in Table 1.

**FIGURE 1:**
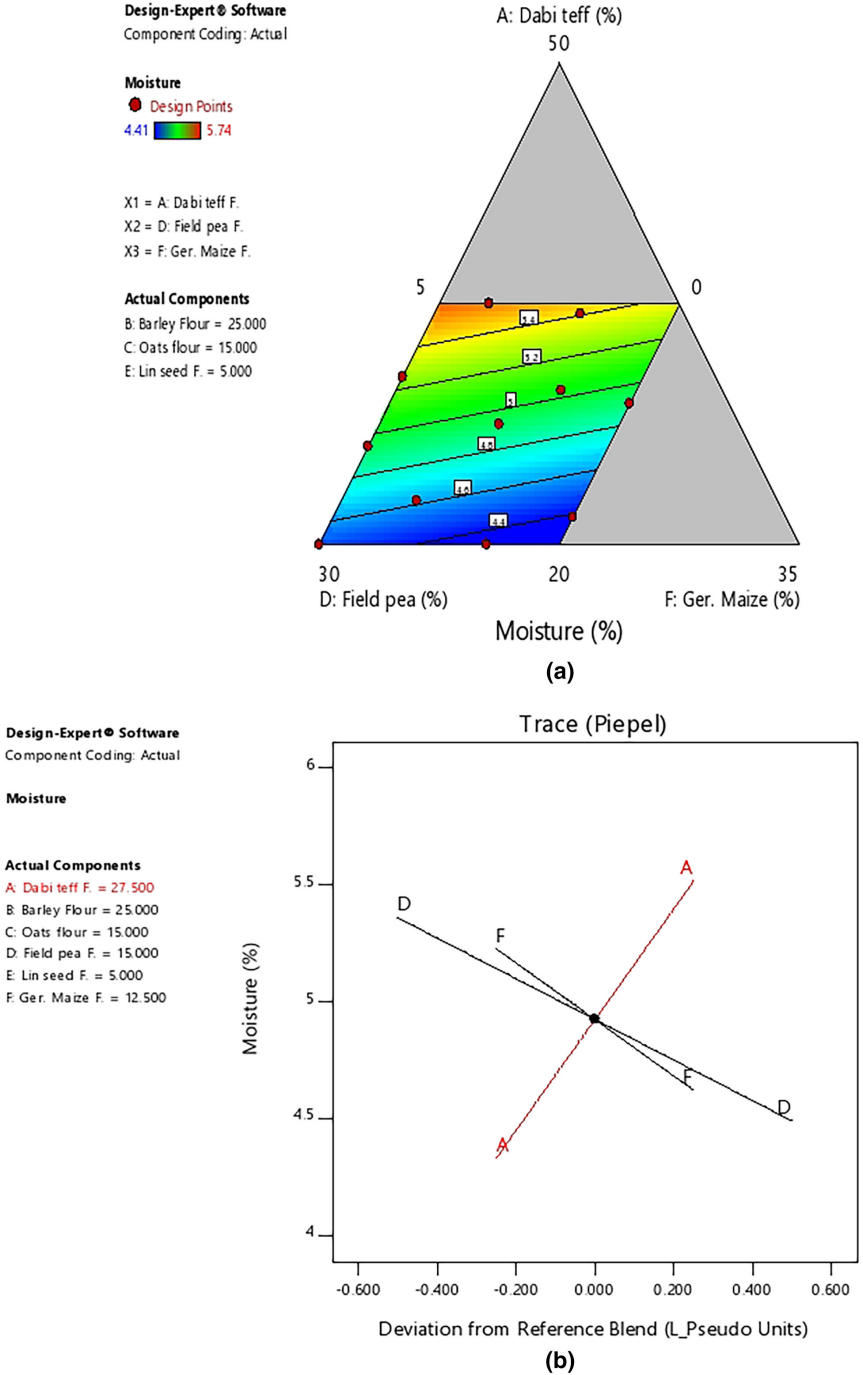
Model graphs showing the effects of the major component ratios on moisture content. (a) 2D contour plot. (b) Trace plots.

**FIGURE 2:**
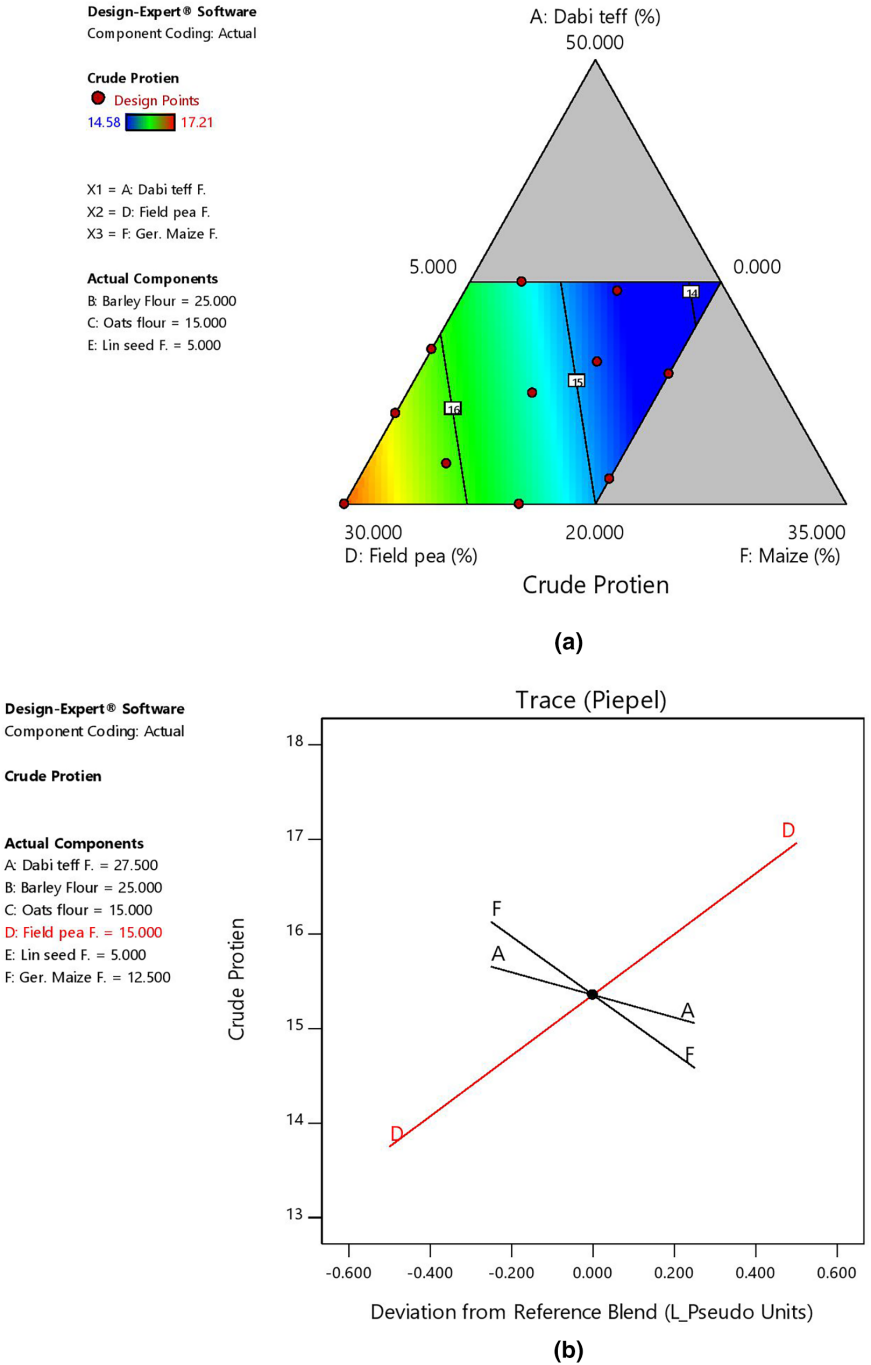
Model graphs showing the effects of the major component ratios on protein content. (a) 2D contour plot. (b) Trace plots.

The observed mean moisture content of the current flour blends was significantly lower (*p* < .05) than the moisture content of the control flour at 6.15%, and the lower moisture content could be attributed to the processing techniques applied. With regard to the effect of component ratio variation in the blends, there was an increase in the moisture content with an increase in the ratio of *dabi* teff flour in the blends, as shown by the 2D contour and trace plots (Figure 1a,b). The observed protein content in the blends was 1.4 to 1.6 times higher than that of the control flour, which was found to be significant at *p* < .05. With regard to the effect of component ratio variation among the blends, there was a linear increasing effect of protein content with an increased ratio of field pea, which could be attributed to the naturally higher protein contents in pulses/legumes foods. It could be observed from Table 6 that at the highest proportion of field peas at 29.98%, the protein content was at the highest value at 17.21% (B4), whereas it was the lowest at 14.58% (B11), where the ratio of field peas was lower at 10.08% in the blends. On the contrary, there was a decreasing effect of protein content as *dabi* teff and germinated maize flour proportions increased, as shown by 2D contour and trace plots (Figure 2a,b).

**FIGURE 3:**
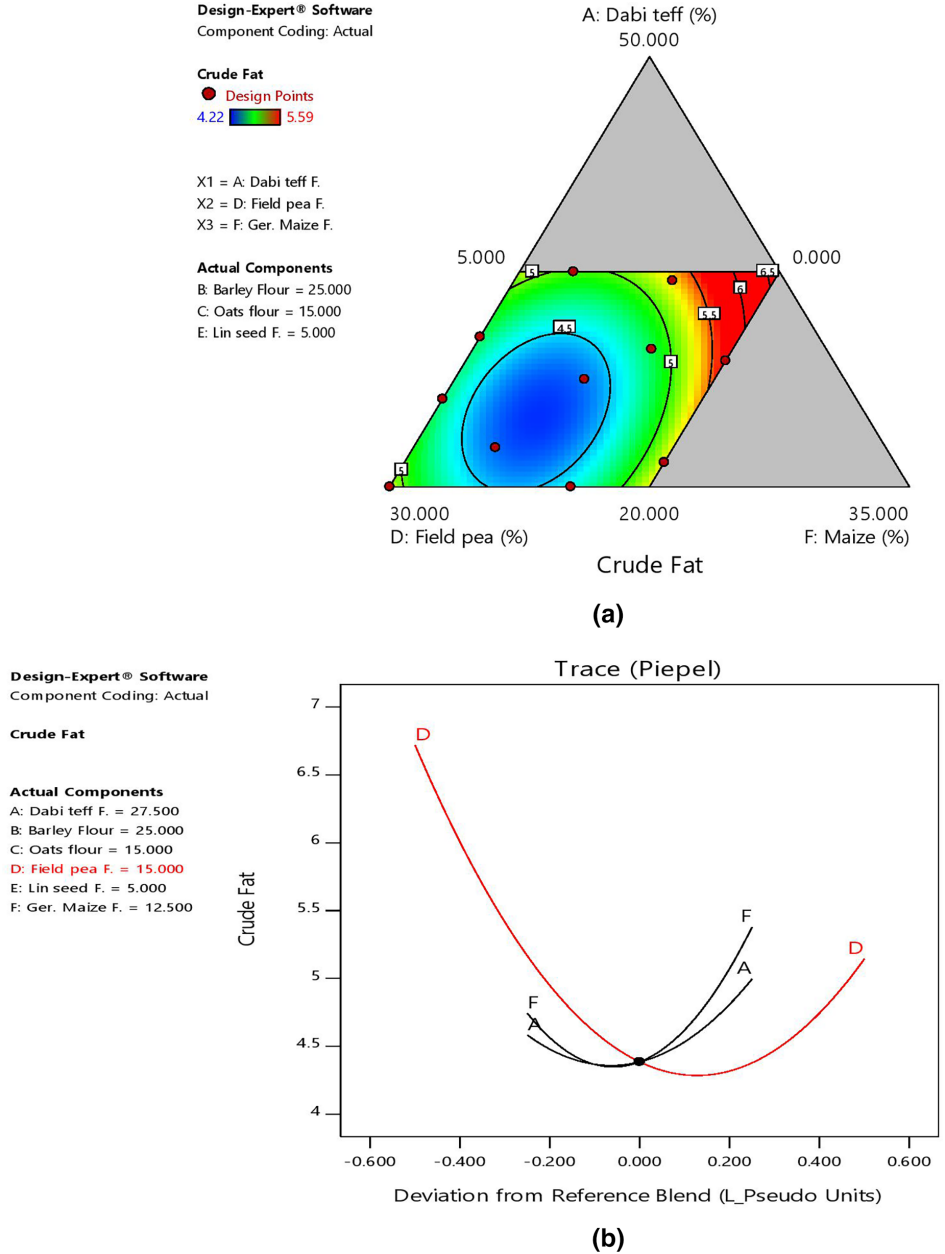
Model graphs showing the effects of the major component ratios on fat content. (a) 2D contour plot. (b) Trace plots.

The mean fat content of the blends at 4.91% was slightly higher than that of the control flour at 3.78%. It had shown the highest fat content at 5.59% (B5) and the lowest at 4.22% (B1) (Table 6). As far as the effect of component ratio variation is concerned, fat content was majorly determined by the field pea and germinated maize ratios in the blends as shown by 2D contour and trace plots (Figure 3a,b).

**FIGURE 4:**
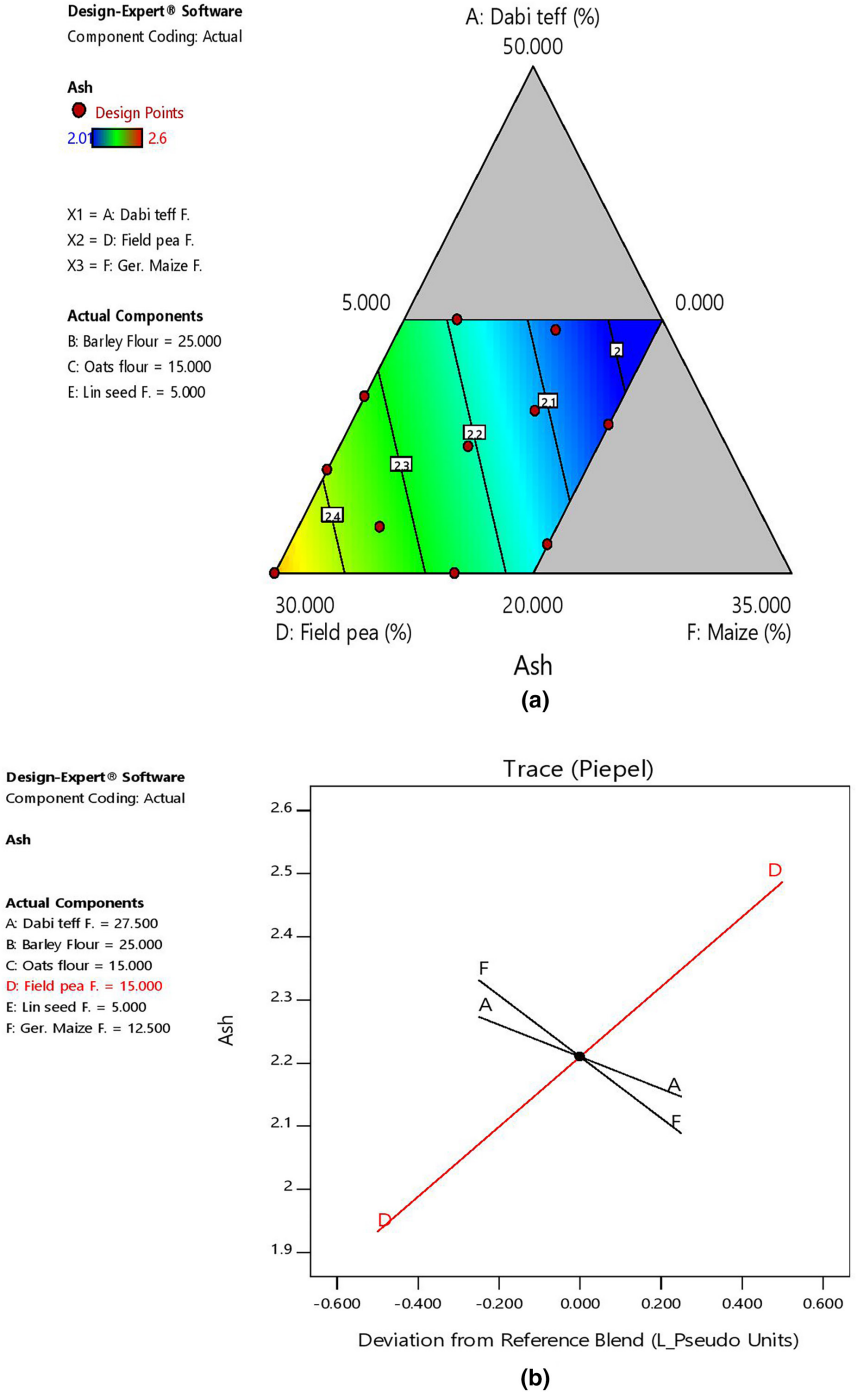
Model graphs showing the effects of the major component ratios on ash content. (a) 2D contour plot. (b) Trace plots.

The mean ash content of the blends at 2.23% was similar to that of the control flour at 2.24%, with the highest at 2.60% (B4) and the lowest at 2.01% (B11). Regarding the effect of component ratio variation, there was a linear increasing effect of ash content with an increased ratio of field pea. On the contrary, there was a decreasing effect of ash content as *dabi* teff and germinated maize flour proportions increased in the blends, as shown by 2D contour and trace plots (Figure 4a,b).

**FIGURE 5:**
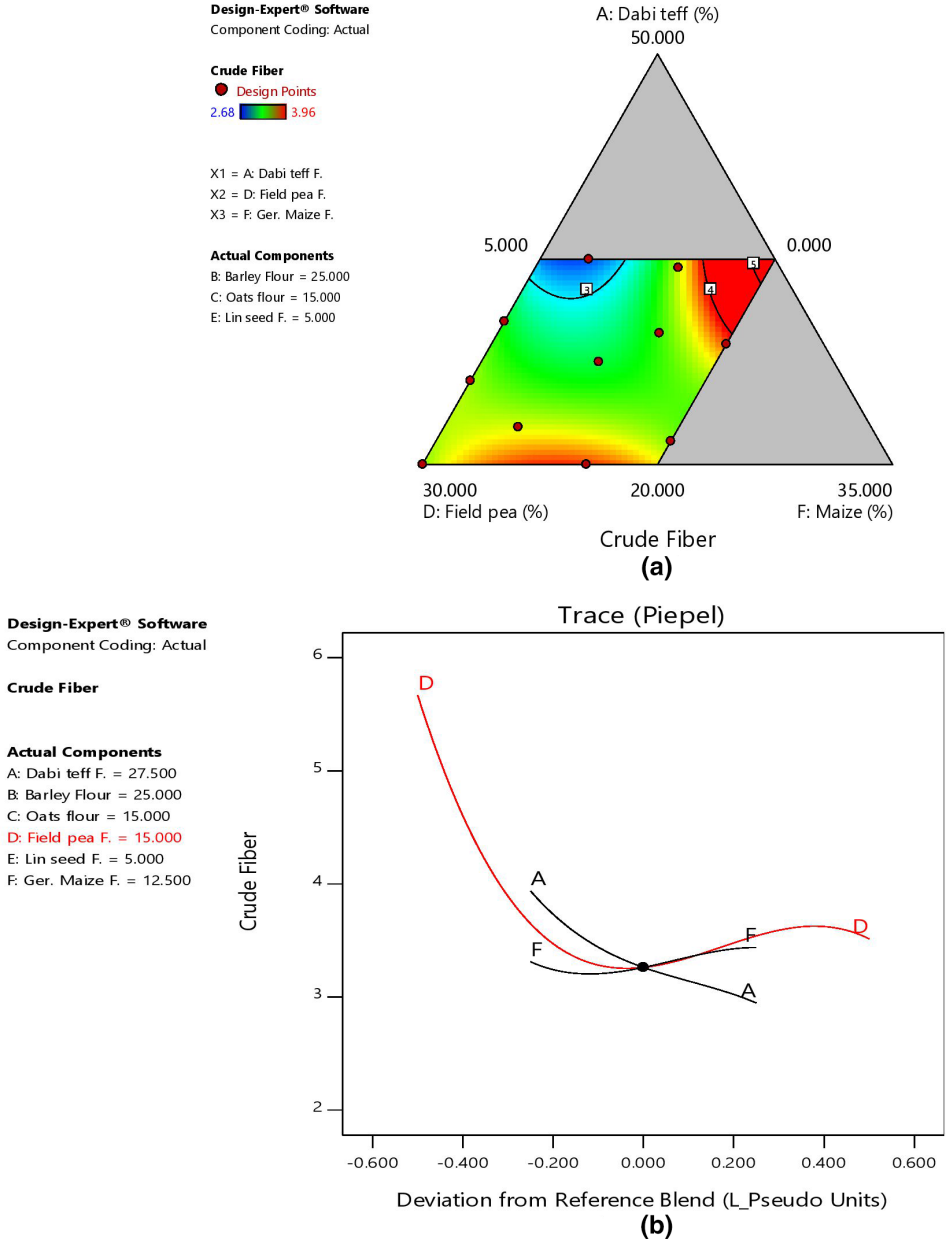
Model graphs showing the effects of the major component ratios on fiber content. (a) 2D contour plot. (b) Trace plots.

The mean fiber content of the blends at 3.48% was slightly higher than that of the control at 2.65%, with the highest and lowest values at 3.96% (B8) and 2.68% (B6), respectively (Table 6). Field pea and germinated maize had a major effect on determining the fiber content among the blends. As field pea and germinated maize ratio increased, fiber content also increased in the blends, as shown by 2D contour and trace plots (Figure 5a,b).

**FIGURE 6:**
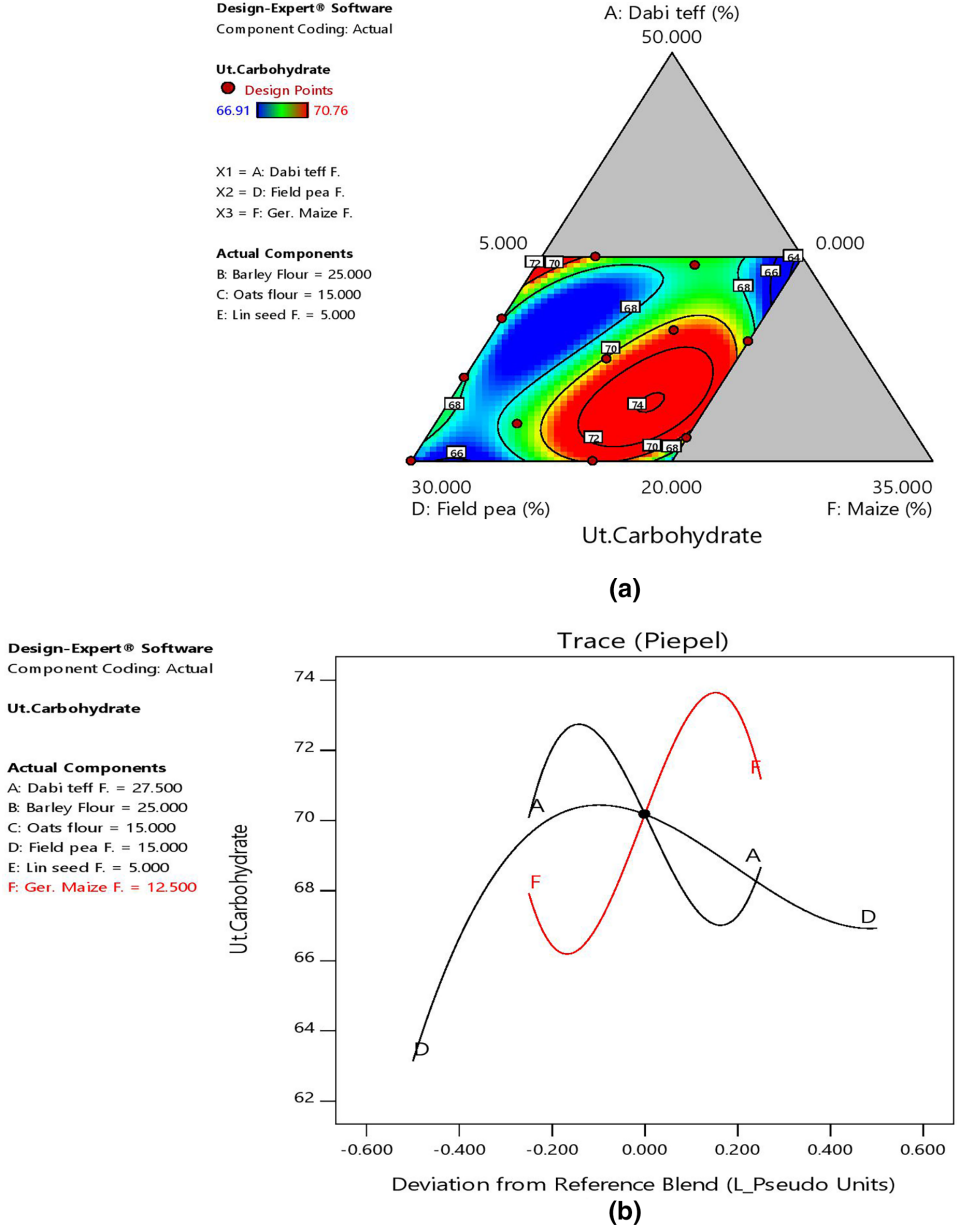
Model graphs showing the effects of the major component ratios on carbohydrate content. (a) 2D contour plot. (b) Trace plots.

The mean carbohydrate content of the blends at 68.99% was slightly lower than that of the control at 74.41%, which could be attributed to barley and maize, from which the control was constructed, with the lowest and highest values at 68.08% (B3) and 70.76% (B11), respectively (Table 6). There was an increasing effect of carbohydrates as the ratio of *dabi* teff and germinated maize flours increased in the blends, as shown by 2D contour and trace plots (Figure 6a,b), which could be attributed to cereals containing a higher carbohydrate content than legumes.

**FIGURE 7:**
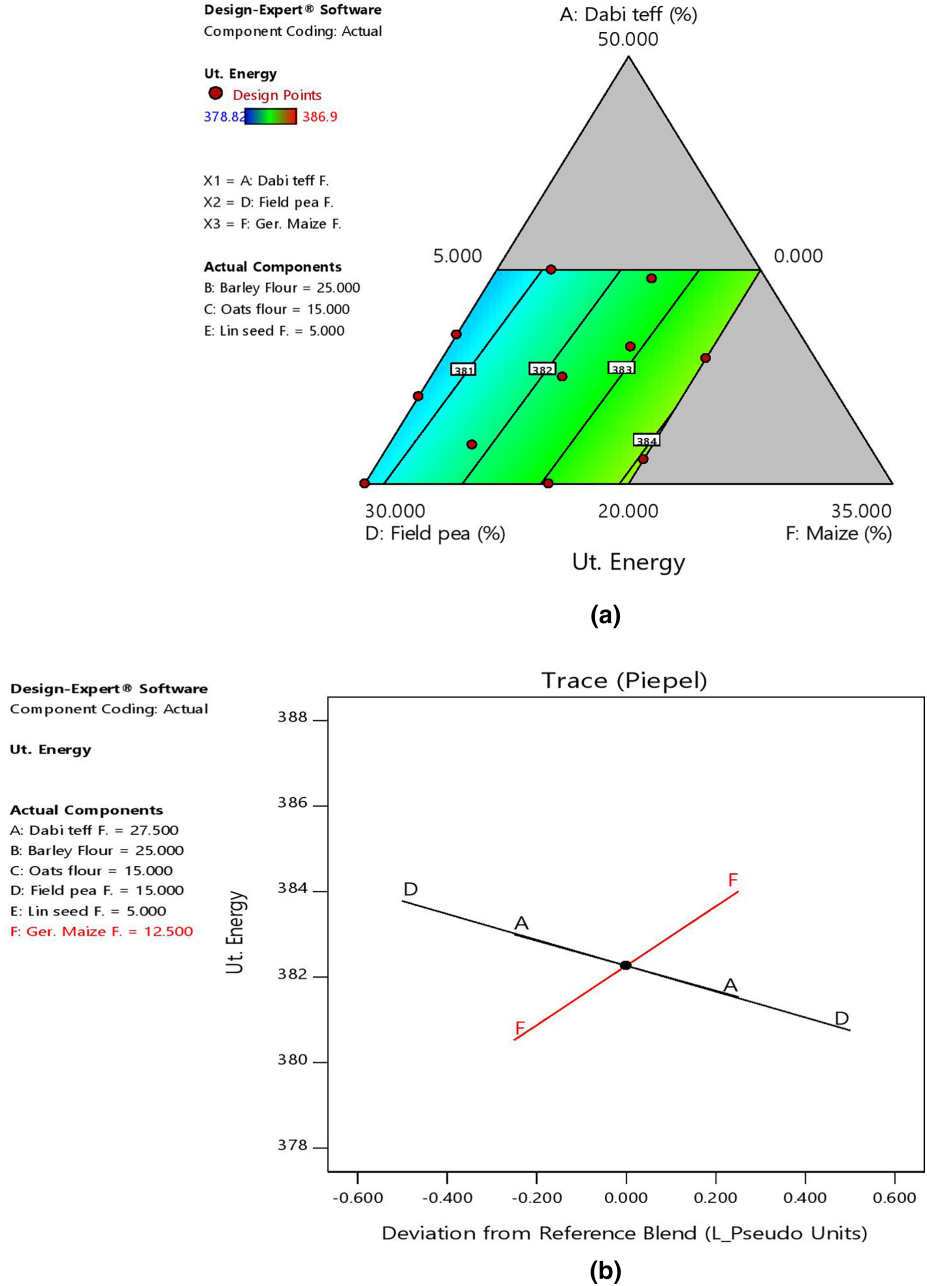
Model graphs showing the effects of the major component ratios on energy content. (a) 2D contour plot. (b) Trace plots.

The mean energy contents of the blends at 382.14 kcal/100 g were significantly higher (*p* < .05) than the control flour at 374.74 kcal/100 g, with its highest and lowest values at 386.9 kcal/100 g (B11) and at 378.82 kcal/100 g (B1) (Table 6). Regarding the effect of component ratio variation in the blends, there was a linear increasing effect in energy content with an increased ratio of germinated maize, and this could be due to the higher energy in cereals. On the other hand, there was a decreasing effect on energy content as *dabi* teff and field pea flour ratios increased in the blends, as shown by 2D contour and trace plots (Figure 7a,b).

### 3.3 Effects of blending ratios on sensory attributes of the complementary porridges

**FIGURE 8:**
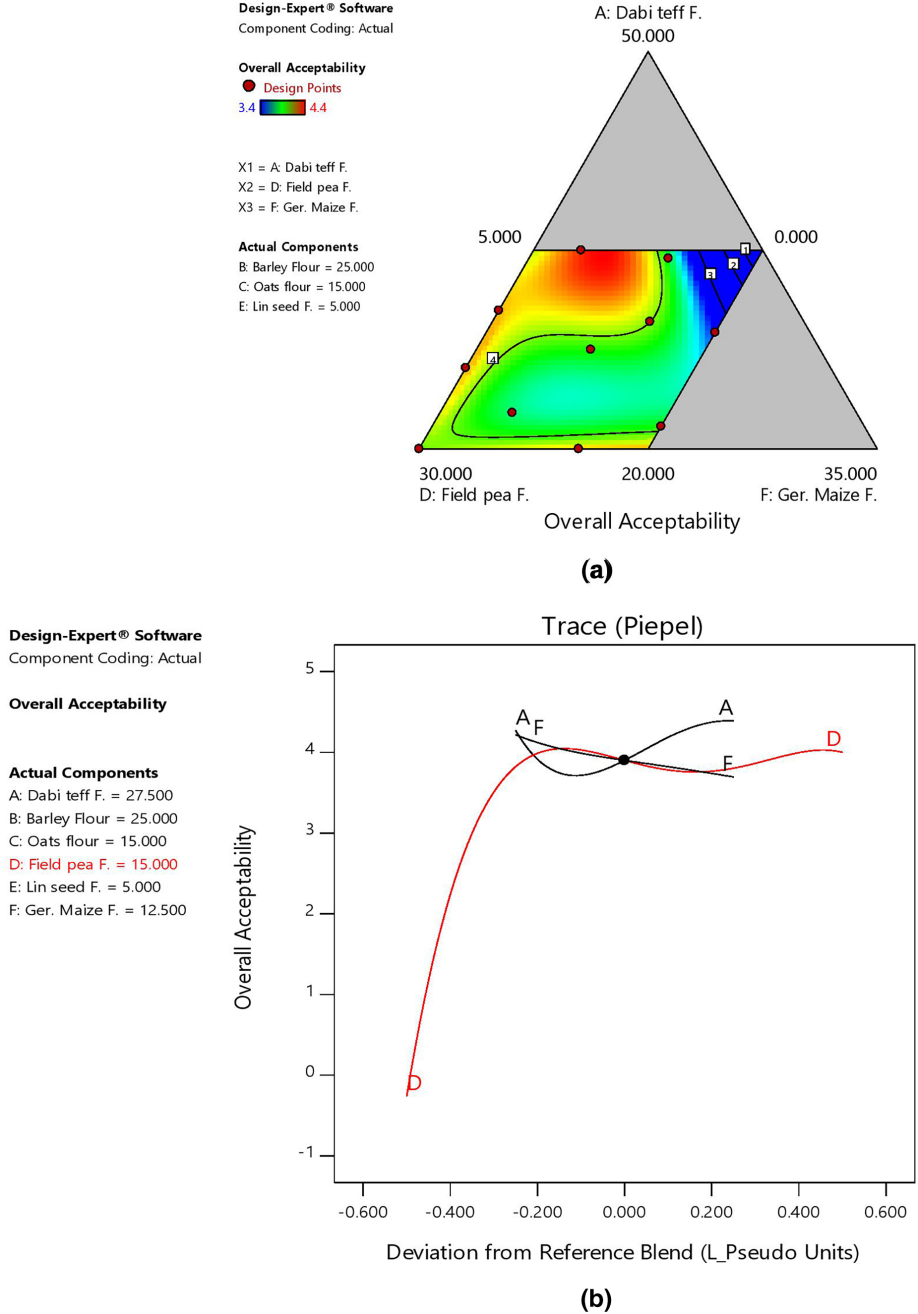
Model graphs showing the effects of the major component ratios on the overall sensory acceptability score. (a) 2D contour plot. (b) Trace plots.

In the present study, the mean values of sensory evaluation scores were significantly different (*p* < .05) among the blends (as affected by component ratio variations) and ranged as 3.4–4.8 for color, 2.8–4.8 for aroma, 3.0–4.4 for taste, 3.2–4.5 for mouthfeel, and 3.4–4.4 for overall sensory acceptability, respectively. The control porridge had 3.5, 4.2, 3.8, 3.9, and 3.4 for color, aroma, taste, mouthfeel, and overall acceptability, respectively (Table 7). The overall sensory acceptability score of the blended porridges was higher (liked much) than that of the control, with the highest and lowest scores at Bp6 (4.4) and Bp5 (3.4), which might be due to the multiple grains and the processing technique applied, which imparted good flavor and aroma to the newly formulated porridges. Regarding the effect of component ratio variation, there was a linear increasing effect in the overall sensory acceptability score with an increased proportion of field pea and *dabi* teff flours, while it showed a negative relationship with germinated maize flour in the blends, as shown by 2D contour and trace plots (Figure 8a,b).

**TABLE 7:** Effects of blending ratios on sensory evaluation of porridges prepared from the blended complementary flours.

Porridge from formulations	Mixture components ratio (%)						Sensory attributes				
	DTF	BF	OF	FPF	LSF	GMF	Color	Aroma	Taste	Mouth feel	OA
BP1	27.50	25	15	15.00	5	12.50	3.5 ± 0.97^a^	3.8 ± 0.92^a^	3.4 ± 0.52^a^	3.8 ± 1.32^a^	3.9 ± 0.74^a^
BP2	26.123	25	15	23.877	5	5.00	3.7 ± 1.06^a^	3.7 ± 0.82^a^	3.4 ± 1.17^a^	4.2 ± 0.79^a^	4.2 ± 1.05^a^
BP3	30.457	25	15	19.543	5	5.00	3.9 ± 0.57^a^	3.3 ± 0.82^a^	3.2 ± 0.92^a^	3.6 ± 0.84^a^	4.2 ± 1.08^a^
BP4	20.00	25	15	29.988	5	5.012	3.4 ± 0.82^a^	3.6 ± 0.82^a^	3.4 ± 0.70^a^	3.3 ± 1.20^a^	4.0 ± 0.94^a^
BP5	28.793	25	15	6.207	5	20.00	4.0 ± 1.2^a^	4.8 ± 1.02^b^	4.1 ± 1.10^a^	4.1 ± 0.99^a^	3.4 ± 0.82^a^
BP6	35.00	25	15	11.884	5	8.116	4.8 ± 0.74^b^	4.0 ± 0.99^a^	4.2 ± 0.92^a^	4.0 ± 0.92^a^	4.4 ± 0.92^b^
BP7	21.705	25	15	13.295	5	20.00	4.0 ± 1.35^a^	4.2 ± 0.92^a^	3.8 ± 1.14^a^	4.2 ± 0.57^a^	3.9 ± 0.99^a^
BP8	34.385	25	15	6.476	5	14.139	4.7 ± 0.99^c^	4.4 ± 0.52^a^	4.1 ± 0.95^a^	4.2 ± 1.14^a^	3.8 ± 0.52^a^
BP9	20.00	25	15	19.540	5	15.460	4.1 ± 0.57^a^	3.8 ± 1.03^a^	3.9 ± 0.88^a^	4.5 ± 0.95^a^	4.3 ± 1.16^a^
BP10	22.735	25	15	22.515	5	9.750	4.0 ± 0.82^a^	2.8 ± 0.88^a^	3.0 ± 0.94^b^	3.2 ± 1.32^b^	3.8 ± 1.05^a^
BP11	29.604	25	15	10.076	5	15.320	4.3 ± 0.62^a^	4.4 ± 0.70^a^	4.4 ± 0.97^a^	4.2 ± 12^a^	4.0 ± 1.03^a^
						Average	4.04	3.89	3.72	3.94	3.99
Control porridge	0	80				20	3.5 ± 1.25^a^	4.2 ± 0.63^a^	3.8 ± 0.63^a^	3.9 ± 0.88^a^	3.4 ± 0.63^a^

*Note*: Values are means ± Standard deviation of the sensory evaluation score. The values in the same column followed by different superscript letters are significantly different at *p* < .05.

Abbreviations: BF, barley flour; BP1–BP11, porridges of the coded formulations; DTF, dabi teff flour; FPD, field pea flour; GMF, germinated maize flour; LSF, linseed flour; OA, overall acceptability; OF, Oats flour.

## 4 DISCUSSION

The ‘novelty’ of this study would be the incorporation of *dabi* teff flour into the blends containing high iron (86.5 mg/100 g) and linseed, which is a leading source of α‐linolenic acid, omega‐3 polyunsaturated fatty acids, that would make the blends super.

In agreement with the present finding, Fikiru et al. (2016) reported that the protein content ranged from 13.0–18.5% in the blends of maize, pea, and malted barley flours, where protein content increased with an increasing ratio of pea and decreased with increasing maize proportion in the blend. Additionally, Mezgebo et al. (2018) reported that increasing proportions of legumes/pulses in food formulations increase the protein content. Mariam (2005) reported that complementary food products from cereal‐legume combinations of two or more components have better protein (overall nutritive value) than products from a single plant food. Such a phenomenon can be well explained by the Food and Agriculture Organization and the World Health Organization (FAO/WHO, 1991) codex alimentarius commission on complementary feeding guidelines. The guidelines describe that mixtures of cereals, legumes, and pulses/oilseed can constitute an appropriate source of nutrients and energy, essential fatty acids, and limiting amino acids with many functional and health benefits, as well as improved organoleptic characteristics.

Fikiru et al. (2016) reported lower fat content that ranged from 1.8%–2.51% from the blends of maize, pea, and malted barley flours, while the report agreed with the present finding that fat content increased with an increasing maize flour ratio in the blends. It is a fact that when non/lower fat‐containing components are added to a relatively higher fat‐containing component, it most likely reduces the concentration of fat in the total sum of the mixture. This is because it is certain that fat content (any response) is a function of a ratio in a mixture.

The current finding was lower than the report by Fikiru et al. (2016) that the ash content in their report ranged from 1.5%–2.5% from the blends of maize, pea and malted barley flours and there was an increase in ash content as an increased proportion of pea in the blends which agreed with the current observation.

The present findings agree with many reports that state that adding legumes to food products increases ash content. Gibson et al. (2006) supported the addition of legumes to cereals, which could lead to higher ash and mineral content. In agreement with the present finding, Fikiru et al. (2016) reported that the fiber content of blends of maize, pea, and malted barley flours ranged between 3.1% and 4.1% and increased with increasing amounts of pea and maize flour.

Inconsistent with the present finding, Fikiru et al. (2016) reported that the carbohydrate contents of complementary flour from maize, pea, and malted barley ranged from 68.9%–74.1% and there was an increase in carbohydrate content with an increasing maize flour ratio. However, our finding was slightly higher than the report by Mezgebo et al. (2018), which found that carbohydrate content ranged from 55.43% to 69.68% for a complementary porridge formulation made from red teff, malted soybean flour, and papaya fruit powder and an increase in carbohydrates with an increased red teff ratio.

Furthermore, our study agreed with the report of Tadesse et al. (2018) that the component variation of bulla, pumpkin, and germinated amaranth had no predictive effect in describing the energy contents of complementary blends. The determined mean value of energy content in the current blends was amenable to the report by Mezgebo et al. (2018) that the energy content of the complementary porridge formulation from red teff, malted soybean flour, and papaya fruit powder ranged from 376.30 to 385.56 kcal/100 g and increased with an increase in malted soya flour. However, Fikiru et al. (2016) reported a lower energy content of complementary flour from maize, pea, and malted barley, ranging from 364.4 to 371.0 kcal/100 g, and energy content increased with an increase in malted barley.

The finding of the overall sensory acceptability score in the present study was lower than the report by Mezgebo et al. (2018), which found that the overall sensory acceptability score ranged from 4.84 to 4.97 for complementary porridge formulations made from red teff, malted soybean flour, and papaya fruit powder and it was accounted for malted soya flour ratio in its formulations.

## 5 CONCLUSION

In most developing countries, complementary foods are monotonous and are characterized by low protein and energy density, which is an immediate cause of protein‐energy malnutrition in children. Foods from multiple grains at some defined mixing ratios are more likely to contain increased and multiple or essential nutrients than foods from a single (mono) grain. The present study had shown that the blending ratio variation of the various ingredients (dabi teff, field pea, and germinated maize, with the other constant ingredients) had a significant effect on the macronutrients and the sensory attributes of the mixture except the fat content. An increase in the ratio of field pea significantly increased the protein, ash, fiber, and energy content of the blends, while an interactive effect between field pea and *dabi* teff flours had a synergetic effect on increasing the overall sensory acceptability. Such effects can provide the basis for optimizing the mixture ratios to develop a wholesome product that can be used to combat protein‐energy malnutrition among children. We suggest conducting and examining the effect of combined and varying processing conditions on nutritional and anti‐nutritional contents, as well as maintaining the original quality of the flour against aflatoxin, rancidity, and microbial safety, as a future recommendation.

## AUTHOR CONTRIBUTIONS

**Diriba Chewaka Tura:** Conceptualization (lead); data curation (lead); formal analysis (lead); funding acquisition (lead); investigation (lead); methodology (lead); project administration (lead); resources (equal); software (lead); supervision (lead); validation (lead); visualization (lead); writing – original draft (lead); writing – review and editing (lead). **Tefera Belachew:** Conceptualization (lead); data curation (lead); formal analysis (equal); funding acquisition (equal); investigation (lead); methodology (lead); project administration (lead); resources (equal); software (lead); supervision (lead); validation (equal); visualization (equal); writing – original draft (equal); writing – review and editing (lead). **Dessalegn Tamiru:** Conceptualization (lead); data curation (lead); formal analysis (equal); funding acquisition (equal); investigation (equal); methodology (equal); project administration (lead); resources (equal); software (equal); supervision (equal); validation (lead); visualization (lead); writing – original draft (lead); writing – review and editing (lead). **Kalkidan Hassen Abate:** Conceptualization (lead); data curation (lead); formal analysis (equal); funding acquisition (equal); investigation (equal); methodology (equal); project administration (lead); resources (equal); software (equal); supervision (lead); validation (lead); visualization (equal); writing – original draft (equal); writing – review and editing (equal).

## CONFLICT OF INTEREST STATEMENT

The authors declare no conflict of interest.

## ETHICS STATEMENT

Ethical clearance for the sensory analyses was obtained from the Institutional Review Board (IRB) of Jimma University, Institute of Health Science, Ethiopia (Ref No. IHRPGS/395/2019).

## CONSENT

Informed verbal consent was obtained from each of the mother's sensory panelists.

## Data Availability

All supportive data are contained within the article in tables and figures.
